# Innovations to Dementia Care: Advancing Equity, Communication, and Preparedness Across Diverse Contexts

**DOI:** 10.1093/geroni/igaf122.417

**Published:** 2025-12-31

**Authors:** Jing Wang, Eunjung Ko

## Abstract

As dementia care evolves to meet the needs of a growing and diverse aging population, innovative strategies are required to improve accessibility, cultural responsiveness, communication, and preparedness. This symposium brings together global perspectives to explore how dementia care can be optimized across different settings and populations. The first presentation provides an updated scoping review of rural palliative dementia care, identifying challenges and strategies to improve person-centered support in resource-limited areas. The second presentation examines culturally adapted interventions for Chinese and Korean American caregivers, showcasing strategies used in the New York University Caregiver Intervention—Enhanced Support (NYUCI-ES) to enhance engagement, support, and care outcomes. The third presentation explores Augmentative and Alternative Communication (AAC) in dementia care, synthesizing research on communication aids and identifying critical gaps in integration and real-world application. The final presentation focuses on disaster preparedness among Chinese American family caregivers, analyzing how caregiving-related characteristics, cultural beliefs, and cognitive appraisals influence emergency planning. This symposium highlights the need for holistic, person-centered, and contextually relevant dementia care models. By addressing rural access barriers, cultural tailoring, communication challenges, and emergency preparedness, these studies provide valuable insights into advancing dementia care across diverse settings. Attendees will gain a deeper understanding of how interdisciplinary, community-driven, and culturally sensitive approaches can enhance dementia caregiving worldwide.

